# Bone marrow mesenchymal stem cell-derived exosomes promote rotator cuff tendon-bone healing by promoting angiogenesis and regulating M1 macrophages in rats

**DOI:** 10.1186/s13287-020-02005-x

**Published:** 2020-11-25

**Authors:** Yao Huang, Bing He, Lei Wang, Bin Yuan, Hao Shu, Fucheng Zhang, Luning Sun

**Affiliations:** Sports Medicine Center, Affiliated Hospital of Nanjing University of Chinese Medicine, Nanjing, 210029 China

**Keywords:** Exosome, Shoulder, Rotator cuff, Tendinosis, Bone mesenchymal stem cells

## Abstract

**Background:**

Rotator cuff tears (RCTs) often require reconstructive surgery. Tendon-bone healing is critical for the outcome of rotator cuff reconstruction, but the process of tendon-bone healing is complex and difficult. Mesenchymal stem cells (MSCs) are considered to be an effective method to promote tendon-bone healing. MSCs have strong paracrine, anti-inflammatory, immunoregulatory, and angiogenic potential. Recent studies have shown that MSCs achieve many regulatory functions through exosomes. The purpose of this study was to explore the role of bone marrow mesenchymal stem cell-derived exosomes (BMSC-Exos) in tendon-bone healing.

**Methods:**

Our study found that BMSC-Exos promote the proliferation, migration, and angiogenic tube formation of human umbilical vein endothelial cells (HUVECs). The mechanism by which BMSC-Exos achieve this may be through the regulation of the angiogenic signaling pathway. In addition, BMSC-Exos can inhibit the polarization of M1 macrophages and inhibit the secretion of proinflammatory factors by M1 macrophages. After rotator cuff reconstruction in rats, BMSC-Exos were injected into the tail vein to analyze their effect on the rotator cuff tendon-bone interface healing.

**Results:**

It was confirmed that BMSC-Exos increased the breaking load and stiffness of the rotator cuff after reconstruction in rats, induced angiogenesis around the rotator cuff endpoint, and promoted growth of the tendon-bone interface.

**Conclusion:**

BMSC-Exos promote tendon-bone healing after rotator cuff reconstruction in rats by promoting angiogenesis and inhibiting inflammation.

**Supplementary information:**

The online version contains supplementary material available at 10.1186/s13287-020-02005-x.

## Background

Rotator cuff tear (RCT) is a common cause of shoulder joint pain and movement disorders [1]. Rotator cuff reconstruction surgery is an important method in treating RCT [2]. Currently, more than 270,000 rotator cuff reconstruction surgeries are performed annually in the USA, and the number is increasing [3]. The rate of re-tear after rotator cuff reconstruction can be as high as 20–94% [4, 5]. The most important reason for re-tears is that tendon-bone healing is not ideal [6]. Promoting tendon-bone healing is a key point in treating RCTs and preventing re-tears after reconstruction.

Most RCTs occur at the tendon-bone interface, where hypovascularization reduces oxygen, growth factors, and other metabolically essential nutrients, leading to slow or nonunion of the tendon-bone interface [6]. Therefore, increased vascularization around the tendon-bone interface is essential for promoting rotator cuff tendon-bone healing [7, 8]. In addition, cytokines play an important role in tendon-bone healing. A variety of proinflammatory factors accumulate around the tendon tissue after operative reconstruction. The area of tissue necrosis expands and leads to disorder of the collagen, myxoid degeneration, and suppression of tendon revascularization. The insufficient vascularization reduces the oxygen supply to the cells, limits the production of various growth factors, inhibits cell proliferation, and eventually affects tendon-bone healing [9].

Recently, BMSCs have been reported to promote rotator cuff tendon-bone healing [10–13]. BMSCs are multipotent stem cells with the potential to differentiate into a variety of cells, including osteoblasts, chondrocytes, and adipocytes, and have the potential to regenerate damaged tissue [14]. Studies in several animal models have shown that transplantation of BMSCs into damaged sites increases the regeneration potential of the bone, cartilage, muscles, ligaments, and tendons in vivo [15, 16]. BMSCs are, therefore, ideal for tissue engineering repair.

Exosomes of the extracellular vesicles can act as paracrine and autocrine regulators to conduct intercellular information transmission and thereby regulate the immune response and tissue metabolism [17]. Exosomes secreted by MSCs play an important role in intercellular signal transduction [18], and exosomes can regulate the tissue microenvironment and promote tissue repair and reconstruction [19]. Currently, there is a lack of research on the mechanism of exosomes in the process of tendon-bone healing. The diverse functions of exosomes suggest that BMSC-Exos may be involved in the regulation of tendon-bone healing.

Our study describes the potential development of stem cells and exosomes in sports medicine. We studied the role of BMSC-Exos in tendon-bone healing in vivo and in vitro. The mechanism may be to promote tendon-bone healing by promoting angiogenesis and inhibiting inflammation.

## Materials and methods

All the experimental procedures were performed with the approval of the Experimental Research Institute of the Nanjing University of Chinese Medicine and followed the guidelines of the Institutional Animal Care and Use Committee.

### Animal experiment in vivo

A total of 59 rats (4-week-old Sprague-Dawley (SD) rats, male, 70–100 g) were used for in vivo experiments. Five rats were used in BMSC extraction, and 54 rats were used in rotator cuff reconstruction model building. Rotator cuff reconstruction model included two groups: phosphate-buffered saline (PBS) group and BMSC-Exos group, and each group included 27 rats. Eighteen rats were used in the tissue section analysis (PBS group, 9; BMSC-Exos group, 9). Eighteen rats were used in the angiogenesis analysis (PBS group, 9; BMSC-Exos group, 9). Eighteen rats were used in the biomechanical test analysis (PBS group, 9; BMSC-Exos group, 9). The rats were sacrificed after tissue was obtained.

### Cells and cell culture

The U937 cells and HUVECs were purchased from the Chinese Academy of Sciences Cell Bank (Shanghai, China). The U937 cells were cultured in 1640 medium (Gibco, Grand Island, NY, USA) containing 10% fetal bovine serum (FBS, HyClone, UT, USA) and 1% double antibiotics (streptomycin + penicillin; Gibco). HUVECs (ATCC, Rockville, MD, USA) were cultured with Dulbecco’s modified Eagle medium (DMEM; Gibco) containing 10% FBS and 1% double antibiotics (streptomycin + penicillin; Gibco). Cells were maintained at 37 °C in a humidified atmosphere of 5% CO_2_ and 95% air.

Five rats were selected, and the BMSCs inside their femurs and tibiae were obtained after anesthesia. Isolated BMSCs were incubated with standard media comprising DMEM (Gibco) supplemented with 10% FBS and 1% double antibiotics (streptomycin + penicillin; Gibco). After 3–5 days of incubation (at 80% confluence), cells were re-plated in 10-cm Petri dishes and maintained at 37 °C in a humidified atmosphere of 5% CO_2_ and 95% air (Fig. S1, Fig. S2).

### The model of U937-M0/M1 macrophages

U937 cells were induced into macrophages according to reference [20]. U937 cells were collected by centrifugation and inoculated in a 6-well plate at 8 × 10^5^ cells/well. U937-M0 (inactive type) was obtained using phorbol myristate acetate (PMA, 5 ng/mL, Biyuntian Company). PMA was added in the dark and the cells cultured for 48 h at 37 °C with 5% CO_2_. The medium containing PMA was then replaced, and 20 ng/mL interferon-gamma (IFN-γ, PeproTech, Rocky Hill, NJ, USA) and 100 ng/mL lipopolysaccharide (LPS, Sigma, St. Louis, MO, USA) were added for 24 h to induce and achieve U937-M1 cells (classic activated type).

### Extraction of exosomes

After BMSCs had grown to about 80% fusion, the culture medium was replaced by an exosome-depleted FBS-containing medium (EXO-FBS-250A-1; System Biosciences, Mountain View, CA, USA). The cells were then cultured a further 48 h. The medium was collected and centrifuged at 4 °C at 300×*g* for 10 min and at 2000×*g* for 10 min. After centrifugation, 0.22 μm Steritop™ (Millipore, Billerica, MA, USA) was used to remove the cells and cell debris. After that, the supernatant was transferred to the upper layer of an Amicon ultra-15 spinning Filter Unit (Millipore), and the supernatant liquid was centrifuged at 4000×*g* at 4 °C to about 200 μL. The supernatant was cleaned with PBS twice and then ultra-filtered to 200 μL. The liquid was placed on a 30% sucrose/D_2_O buffer and placed in a sterile Ultra-Clear™ tube (Beckman Coulter, Brea, CA, USA) and centrifuged at 100,000×*g* for 60 min at 4 °C (Sorvall, Avanti J-26XP, fixed-angle rotor; Beckman Coulter). The fraction containing the BMSC-Exos was recovered using an 18-G needle, diluted in PBS, and centrifuged at 4000×*g* at 4 °C in centrifugal filter units until the final volume reached 200 μL. BMSC-Exos were stored at − 80 °C. The protein content of BMSC-Exos was measured by the bicinchoninic acid assay (BCA; Thermo Fisher, Waltham, MA, USA). A microplate reader (ELx800, BioTek, USA) was used to measure the absorbance at a wavelength of 562 nm.

### Immunofluorescence and immunohistochemistry

For the immunofluorescence assays, briefly, the paraffin sections were dewaxed, dehydrated, and incubated overnight at 4 °C with anti-CD31 and anti-endomucin (diluted 1:100; Abcam, Cambridge, UK). The secondary antibody (diluted 1:100; Jackson, West Grove, PA, USA) was applied, and the cells were incubated for 1 h in the dark. Finally, the nuclei were counterstained with DAPI for 15 min. The stained cells were photographed using a fluorescence microscope.

For the cell immunofluorescence (Carl Zeiss Microscopy GmbH, Jena, Germany) assays, HUVECs were cultured in a 24-well plate. The cells were fixed with 4% paraformaldehyde and incubated with 0.5% Triton X-100 (Sigma) and then blocked with goat serum (Biyuntian Company, China) for 1 h. The cells were then incubated overnight at 4 °C with the primary antibody (anti-YAP1, diluted 1:100; Abcam). The secondary antibody (diluted 1:200; Jackson, West Grove, PA, USA) was applied, and the cells were incubated for 1 h in the dark. Finally, the nuclei were counterstained with DAPI for 15 min. The stained cells were photographed using a fluorescence microscope.

For the immunohistochemistry assays, the paraffin sections were dewaxed, dehydrated, and incubated overnight at 4 °C with anti-type I collagen (Col I), anti-type II collagen (Col II), and anti-CD86 (diluted 1:100; Abcam). After the primary antibody was removed, the secondary antibody (diluted 1:100, Thermo Fisher) was added, and the sections were incubated for 1 h at room temperature. The stained cells were developed with diaminobenzidine and counterstained with hematoxylin.

### Safranin and fast green staining

After the shoulder joint was fixed and decalcified, the shoulder was embedded in paraffin, sliced at 5 μm in the coronal position, and hydrated after dewaxing. For Fast Green staining, the slices were placed in the Fast Green dye liquid for 5–10 min. Excess dye was washed with water until the cartilage became colorless. The slices were then slightly soaked in the differentiation fluid and then washed with tap water. For staining with Safranin, the slices were placed in Safranin dye liquid for 15–30 s and then quickly dehydrated with three cylinders of anhydrous ethanol. For the transparent seal, sections were cleared with xylene for 5 min and then sealed with a neutral gum seal. The cartilage stained red or orange with a green background.

### Western blot analysis

Cells were placed on ice immediately following treatment and washed with ice-cold Hank’s Balanced Salt Solution. Nuclear proteins were prepared using the CellLytic NuCLEAR extraction kit (Sigma-Aldrich). All the wash buffers and final resuspension buffer included a 1× protease inhibitor cocktail (Pierce, Rockford, IL, USA), NaF (5 mM), and Na3VO4 (200 mM). The protein concentration of the lysate was measured using the BCA protein assay kit (Thermo Fisher). Nuclear or total cell proteins were resolved on 8 to 12% SDS-PAGE and transferred by electroblotting to nitrocellulose membranes (Bio-Rad, Hercules, CA, USA). The membranes were blocked in 5% bovine serum albumin reagent (Beyotime, Jiangsu, China) and incubated overnight at 4 °C with primary antibody dilution buffer (the dilution followed the specification; Abcam) and then incubated with horseradish peroxidase-conjugated anti-rabbit IgG (1:5000) for 2 h. Afterward, the membranes were developed using the enhanced chemiluminescence substrate LumiGLO (Millipore, Bedford, MA, USA) and exposed to X-ray film. The bands were analyzed with Gel-Pro Analyzer 4.0 (Bio-Rad, Hercules, CA, USA).

### RNA isolation and quantitative real-time polymerase chain reaction

Primers for quantitative real-time polymerase chain reaction (qRT-PCR) were designed using Primer-BLAST (http://www.ncbi.nlm.nih.gov/tools/primer-blast/). Total RNA from cells was isolated with TRIzol® Reagent (Invitrogen, Carlsbad, CA, USA), and cDNA was synthesized by a first-strand cDNA synthesis kit (Thermo Fisher) according to the manufacturer’s instructions. Quantitative real-time polymerase chain reaction (qRT-PCR) was performed using the iQ5 optical system software (Bio-Rad) with FastStart Universal SYBR Green Master (Recho, Basel, Switzerland) for mRNA quantitation of all referred genes. Relative expression was calculated using the 2^−ΔΔCt^ method normalized to GAPDH (endogenous loading control). The following primers were used: GAPDH, sense (5′-GGAGCGAGATCCCTCCAAAAT-3′), anti-sense (5′-GGCTGTTGTCATACTTCTCATGG-3′); interleukin (IL)-1β, sense (5′-ATGATGGCTTATTACAGTGGCAA-3′), anti-sense (5′-GTCGGAGATTCGTAGCTGGA-3′); TNF-α, sense (5′-CCTCTCTCTAATCAGCCCTCTG-3′), anti-sense (5′-GAGGACCTGGGAGTAGATGAG-3′); IL-6, sense (5′-ACTCACCTCTTCAGAACGAATTG-3′), anti-sense (5′-CCATCTTTGGAAGGTTCAGGTTG-3′); IL-8, sense (5′-ACTGAGAGTGATTGAGAGTGGAC-3′), anti-sense (5′-AACCCTCTGCACCCAGTTTTC-3′); and nitric oxide synthase 2 (NOS 2), sense (5′-AGGGACAAGCCTACCCCTC-3′), anti-sense (5′-CTCATCTCCCGTCAGTTGGT-3′).

### Flow cytometry

U937-M0 cells were incubated with IFN-γ + LPS and BMSC-Exos for 24 h. Cells (1 × 10^6^/tube) were washed twice with PBS and then resuspended in 100 μL PBS before 1 μL FITC-labeled CD86 antibody (BD Biosciences, San Jose, CA, USA) was added. The cells were incubated for 30 min at 4 °C in the dark. Any unbound antibody was washed away by PBS before the cells were resuspended in 300 μL PBS. Flow cytometry (FACSCalibur, BD Biosciences, USA) was used to detect the expression of CD86 on the cell surface.

### Enzyme-linked immunosorbent assay

TNF-α, IL-1β, IL-6, and IL-8 were determined by enzyme-linked immunosorbent assay (ELISA) using a commercially available ELISA set (R&D Systems Inc., Minneapolis, MN, USA). ELISA was performed according to the manufacturer’s instructions. All samples and standards were measured in duplicate.

### Cell viability assay

The Cell Counting Kit-8 (CCK-8; Dojindo, Kumamoto, Japan) was used to evaluate the effects of BMSC-Exos on HUVEC proliferation. BMSC-Exos (100 μg/mL) or the same volume of PBS was added into the culture medium, and the cells were incubated for 0, 24, 48, 72, and 96 h. After incubation, PBS was used to wash cells for 3 times, and CCK-8 solution (10 μL; 1:10 diluted) was added into the fresh culture medium at 37 °C for 2 h. Finally, the optical absorbance for each sample was measured at 450 nm using an absorbance microplate reader (ELx800, BioTek).

Cell proliferation was also measured using the 5-ethynyl-29-deoxyuridine (EdU) assay kit (RiboBio, Guangzhou, China) according to the manufacturer’s instructions. Briefly, cells were seeded into 24-well plates at a density of 2.0 × 10^4^ cells/well and cultured for 24 h before the administration of EDU (50 mM). After, Apollo and DNA stains were added. Finally, proliferation images were acquired and analyzed by fluorescence microscopy (Carl Zeiss Microscopy GmbH, Jena, Germany).

### Tube formation assay

To evaluate angiogenesis induced by BMSC-Exos, HUVECs were inoculated on Matrigel-coated 96-well plates (Matrigel, BD biosciences, CA, USA) at 2 × 10^4^ cells/well. Matrigel was dissolved overnight at 4 °C in advance and placed in each hole upon ice. BMSC-Exos (100 μg/mL) or PBS was added to each hole, and then the cells were cultured for 30 min at 37 °C. The polygonal structures of HUVECs were observed with an optical microscope 6 h after the cells had been placed on the Matrigel. The tube-forming ability was evaluated by measuring the total length and number of tube branches.

### Migration assays

Transwell migration was used to measure the effect of BMSC-Exos on HUVEC migration. HUVECs (5 × 10^4^ cells/hole) were added into the upper chamber of the transwell chamber (Corning, NY, USA; pore size 8 μm). BMSC-Exos (100 μg/mL) or PBS was added in the lower chamber for a 24-h culture. The cells that did not migrate above the filter membrane were gently wiped off using a cotton swab. The cells that migrated to the bottom of the filter membrane were fixed and stained with hematoxylin staining. Five fields were randomly selected under the microscope to calculate the number of migratory cells.

The scratch wound assay was used to evaluate the effect of BMSC-Exos on HUVEC migration. HUVECs were inoculated in a 24-well plate (2 × 10^5^ cells/hole), and after the cells had grown to 90% confluence, a sterile 200-μL pipette tip was used to scratch the cells in the hole vertically. The scratch in each hole was required to be straight, and the scratch in each hole was the same. The removed cells were washed 2 times with PBS, and then 0 or 100 μg/mL BMSC-Exos was added. The cell migration images were recorded 0, 12, and 24 h after the scratch.

### Nitric oxide assay

U937 or M1 macrophages (2 × 10^5^ cells/mL) were placed in the cell culture plate. To observe the nitric oxide (NO) release during the induction of U937-M1 macrophages, BMSC-Exos (100 μg/mL) or equivalent PBS was added to the culture medium 1 h before the addition of IFN-γ and LPS. NO release was analyzed in the supernatant every 24 h for four consecutive days. The Griess Reagent (Promega, USA) mixed with an equivalent volume of culture supernatant was incubated at room temperature for 10 min. The absorbance was determined by spectrophotometry at 540 nm. The sodium nitrite (NaNO_2_) standard curve was used to determine the concentration of NO.

### RCT and reconstruction model building

After successful anesthesia with an intraperitoneal injection of 10% chloral hydrate (0.3 mL/100 g), the shoulder joint was incised laterally and the trapezius muscle was pulled away. The insertion to the humeral head of the supraspinatus was exposed. The supraspinatus insertion was resected. Approximately 2 mm of the distal tendon of the supraspinatus was cut off. Then, the skin was sutured. An RCT in rats is considered to simulate the chronic RCT that frequently occurs in humans [21]. Four weeks later, a 3-0 nonabsorbable Propathene line was used to fix the supraspinatus tendon to the humeral head through the bone tunnels. The subjects were divided into two groups. In the PBS group, PBS was injected into the tail vein, and in the BMSC-Exos group, BMSC-Exos (200 μg of total protein of BMSC-Exos precipitated in 200 μL of PBS) was injected into the tail immediately after suturing.

### Micro-CT analysis of angiogenesis at the reconstruction sites

The micro-CT system (Siemens Inveon PET/CT, Berlin, German) was used to assess vascularity. Vascular networks around the shoulder junction were examined using micro-CT analyses combined with perfusion of a contrast agent. Briefly, the blood vessels were first rinsed with normal saline containing heparin and 4% paraformaldehyde (PFA). Then, using MICROFIL® injection compound (Flow Tech, Inc., Carver, MA) contrast media, a radio-opaque silicone rubber compound containing lead chromate was perfused via the heart. After perfusion, the reconstruction shoulder was removed and scanned using the micro-CT system. The samples were subsequently decalcified for 30 days using a 10% EDTA solution. After complete decalcification, the samples were scanned again to visualize only the vascularization within the callus tissue. Three-dimensional reconstructions were made using the Inveon research workplace (2.0, Berlin, German).

### Biomechanical test

Biomechanical tests were performed 4 and 8 weeks after the surgical reconstruction. Mutilation was performed at the lower end of the humerus. The supraspinatus and its insertion were carefully retained; the other muscles, except for the supraspinatus, were removed. The supraspinatus-humerus structure was obtained. The specimen was immediately placed in a 4% paraformaldehyde solution, and the shoulder joint tissue was firmly fixed on an INSTRON biomechanical tester (Instron, Boston, MA, USA). The instrument was loaded, and a displacement velocity of 5 mm/min was applied to test the maximum tensile load of the supraspinatus tissue. The loading load when the supraspinatus tendon was broken was observed and recorded in detail as the maximum breaking load (N). The linear slope of the load-displacement curve was taken as the stiffness.

### Statistical analysis

The SPSS 19.0 statistical software (SPSS, Inc., Chicago, IL, USA) was used for all statistical analyses. The measurements are presented as the mean ± standard deviation. The data were analyzed using a one-way analysis of variance followed by Bonferroni’s post hoc test for multiple comparisons. *p* < 0.05 was considered to indicate a statistically significant difference.

## Results

### Characterization of BMSC-Exos

**Fig. 1:**
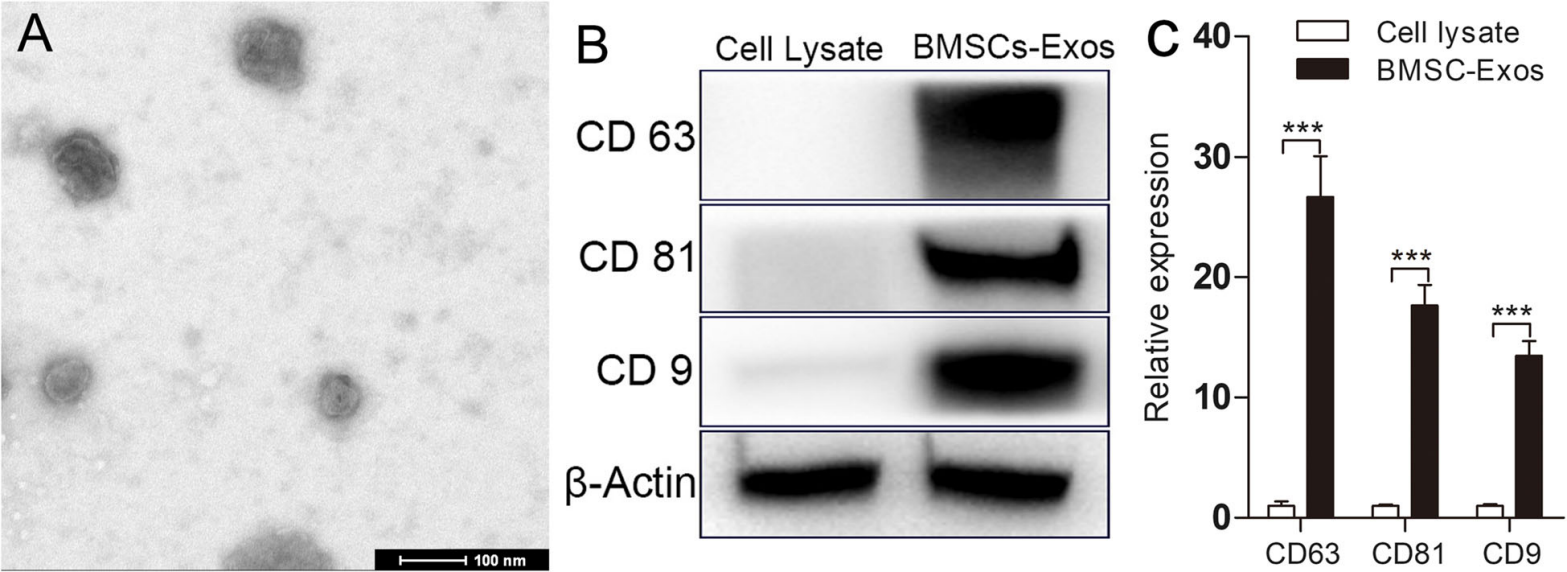
BMSC-Exos were identified by TEM and WB. **a** Morphology observed by TEM. **b** Exosome surface markers detected by Western blot. **c** Quantitative analysis of **b**. Each experiment was independently repeated three times (*p < 0.05; **p < 0.01; ***p < 0.001)

Transmission electron microscope (TEM) and Western blot (WB) were used to identify particles from BMSCs. As shown in Fig. 1a, the particles were circular under TEM and had a diameter of about 30–150 nm. WB was used to identify the exosome-specific phenotypic markers CD9, CD63, and CD81 on the microparticle surface (Fig. 1b, c; Fig. S3). These were consistent with previous reports [22, 23], indicating that the BMSC-Exos particles that we extracted were BMSC-Exos.

### BMSC-Exos promoted proliferation, migration, and angiogenic tube formation in HUVECs

**Fig. 2:**
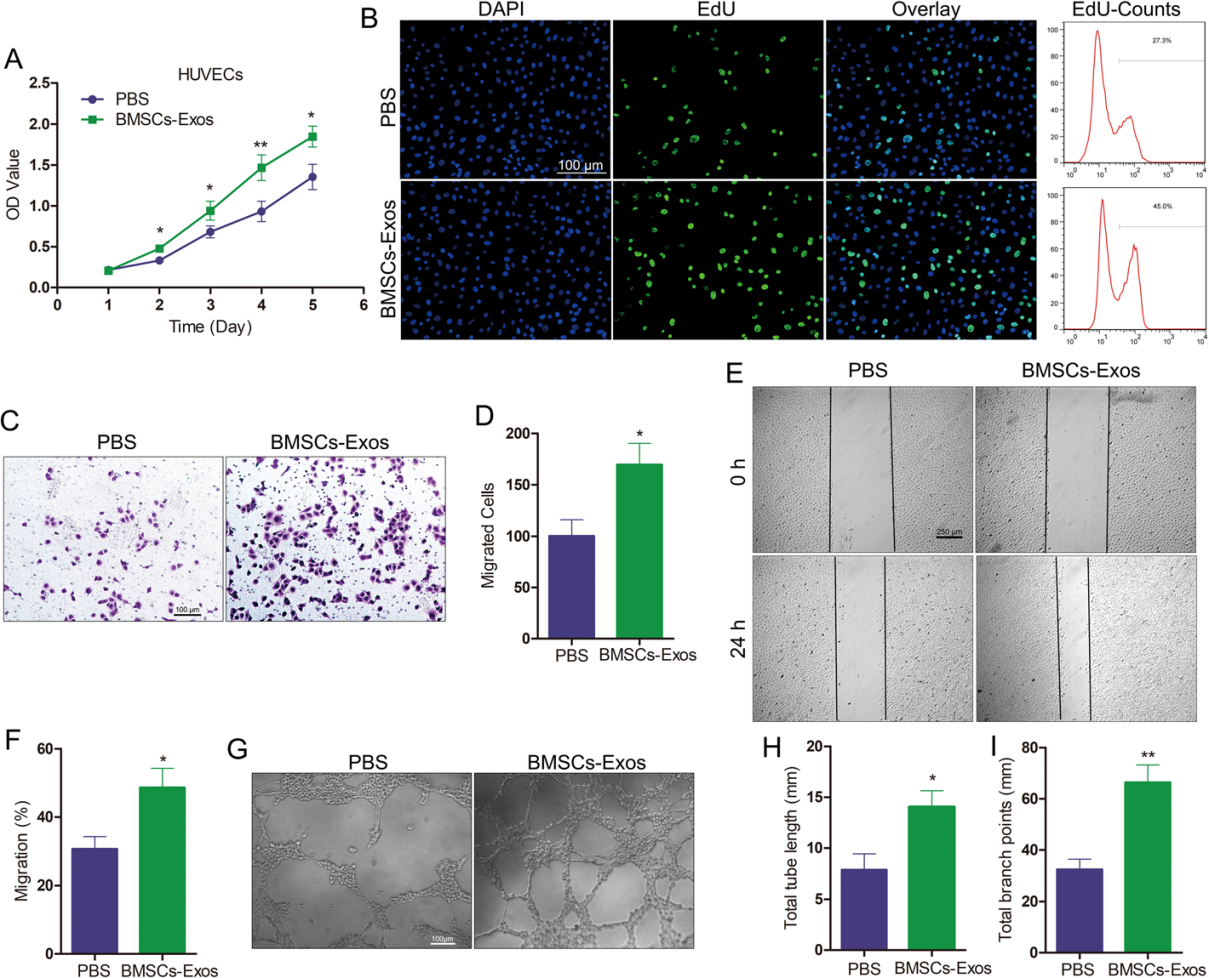
Functional effects of BMSC-Exos on HUVECs. **a**, **b** BMSC-Exos significantly promoted the proliferation of HUVECs, as demonstrated by the Cell Counting Kit-8 (CCK-8) and EdU assays. Scar bar 100 μm. **c** BMSC-Exos significantly promoted the migration of HUVECs as determined by the transwell assay at 24 h. Scar bar 100 μm. **d** Quantitative analysis of the transwell assay. **e** BMSC-Exos significantly promoted the mobility of HUVECs as determined by the scratch wound assay. Scar bar 100 μm. **f** Quantitative analysis of the scratch wound assay. **g** BMSC-Exos significantly enhanced the tube formation ability at 6 h, as determined by the tube formation assay. Scar bar 250 μm. **h**, **i** Quantitative analysis of the tube formation assay. Each experiment was independently repeated three times (*p < 0.05; **p < 0.01; ***p < 0.001)

In order to study the effects of BMSC-Exos on angiogenesis, we first observed the potential effects of BMSC-Exos on HUVECs. BMSC-Exos (100 μg/mL) were added or not added to the culture medium of HUVECs to observe the effect of BMSC-Exos on these cells. The proliferation of HUVECs was detected by the CCK-8 assay. BMSC-Exos promoted the proliferation of the HUVECs (Fig. 2a). The EdU assay also showed that BMSC-Exos promoted the proliferation of HUVECs (Fig. 2b).

In addition, we observed the effect of BMSC-Exos on HUVEC migration and angiogenic tube formation. Transwell experiments showed that incubation with BMSC-Exos promoted HUVEC migration (Fig. 2c, d). The scratch wound confirmed that BMSC-Exos promoted the migration of HUVECs, and the results showed that the application of BMSC-Exos improved the mobility of HUVECs (Fig. 2e, f). After HUVECs began to form capillaries, the length of the tubes and branching points was calculated. As shown in Fig. 2g–i, BMSC-Exos promoted lumen formation at 6 h.

### BMSC-Exos activated the angiogenic signaling pathway in HUVECs

**Fig. 3:**
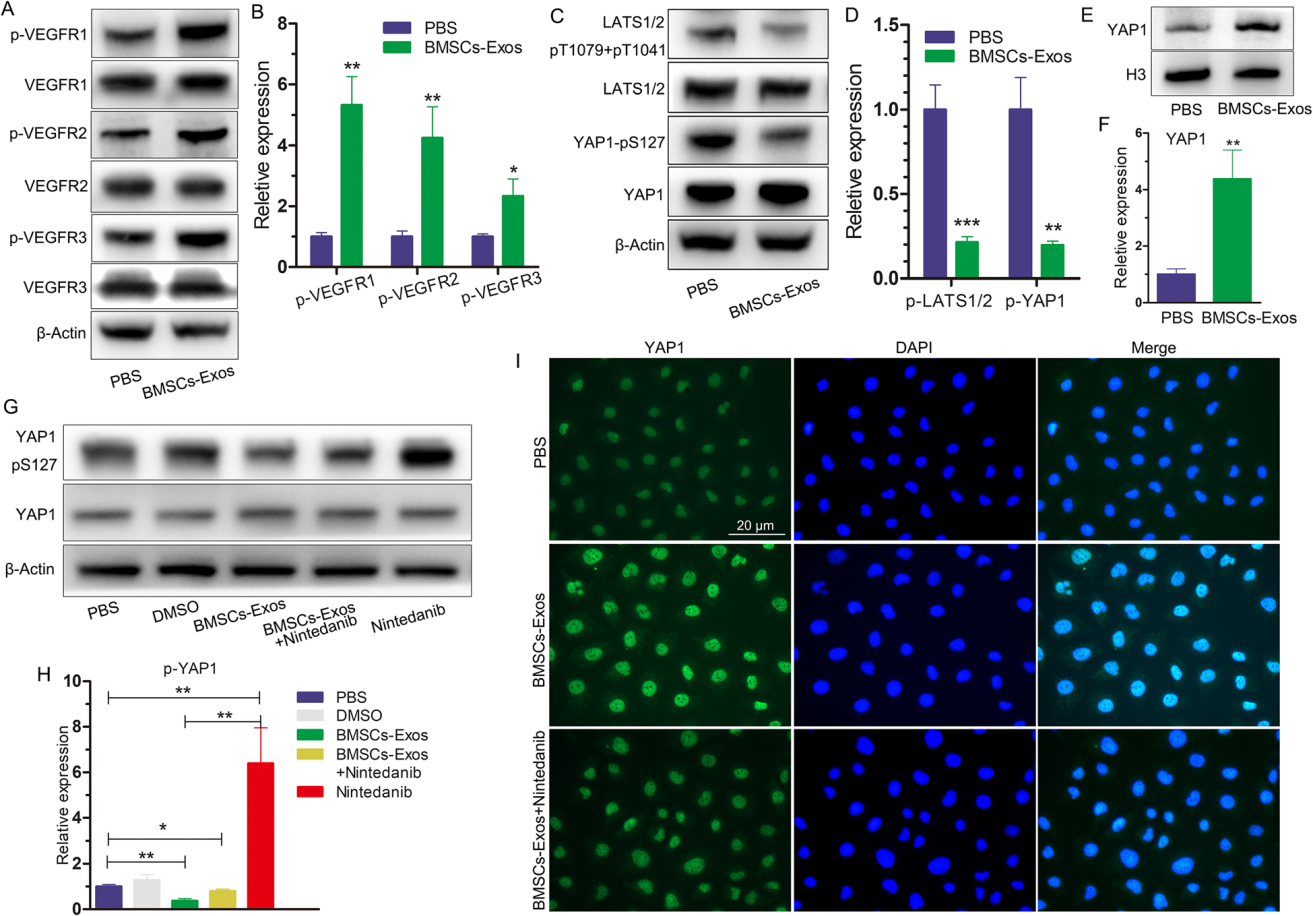
Functional effects of BMSC-Exos on angiogenesis-related signaling pathways in HUVECs. **a** BMSC-Exos promoted the phosphorylation of VEGFR at 12 h, as determined by WB in HUVECs. **b** Quantitative analysis of **a**. **c** BMSC-Exos inhibited the phosphorylation of LATS and YAP1 at 12 h, as determined by WB in HUVECs. **d** Quantitative analysis of **c**. **e** BMSC-Exos increased YAP1 expression in the nucleus at 12 h, as determined by WB in HUVECs. **f** Quantitative analysis of **e**. **g** Effects of BMSC-Exos and nintedanib on YAP1 phosphorylation at 12 h, as determined by WB in HUVECs. **h** Quantitative analysis of **g**. **i** Effects of BMSC-Exos and nintedanib on YAP1 expression at 12 h, as determined by immunofluorescence in HUVECs. Each experiment was independently repeated three times. Scar bar 20 μm

The vascular endothelial growth factor (VEGF) signaling pathway is very important for angiogenesis. VEGF induces vascular endothelial growth factor receptor (VEGFR) phosphorylation and activates the VEGF signaling pathway. We used WB to detect the effect of BMSC-Exos on VEGFR and found that the phosphorylation levels of VEGFR1 and VEGFR2, and to a lesser extent VEGFR3, were increased after BMSC-Exos had been added to the culture medium (Fig. 3a, b). The results show that BMSC-Exos induced the activation of the VEGF signaling pathway.

Recent studies have found that activation of the Hippo signaling pathway is correlated with angiogenesis and that decreased phosphorylation of large tumor suppressor serine/threonine protein kinases (LATS) and Yes-associated protein (YAP1) leads to increased expression of YAP1 in the nucleus and activation of the Hippo pathway [24]. After adding BMSC-Exos into the culture medium, WB detection showed that phosphorylation of LATS1/2 and YAP1 was inhibited (Fig. 3c, d), and that YAP1 expression was increased in the nucleus (Fig. 3e, f). The results of the WB show that BMSC-Exos activated the Hippo signaling pathway.

Phosphorylation of VEGFR can inhibit the phosphorylation of LATS and YAP [25]. To test whether BMSC-Exos inhibits the phosphorylation of LATS and YAP by phosphorylating VEGFR, we added BMSC-Exos and the VEGFR inhibitor nintedanib (MedChemExpress, Monmouth Junction, NJ, USA; 50 nmol/L, dissolved in dimethyl sulfoxide (DMSO)) to the HUVEC culture solution. The final concentration of DMSO in the culture medium was less than 0.1%. WB showed that phosphorylation of YAP1 was inhibited when BMSC-Exos was added, while phosphorylation of YAP1 was increased when only nintedanib was added to the culture medium (Fig. 3g, h). Immunofluorescence showed that BMSC-Exos induced the expression of YAP1 in the nucleus, while nintedanib reduced the expression of YAP1 in the nucleus (Fig. 3i). The results indicate that BMSC-Exos activated the Hippo signaling pathway through the VEGF pathway in the HUVECs.

### BMSC-Exos promoted angiogenesis around the tendon-bone interface of the rotator cuff in rats

**Fig. 4:**
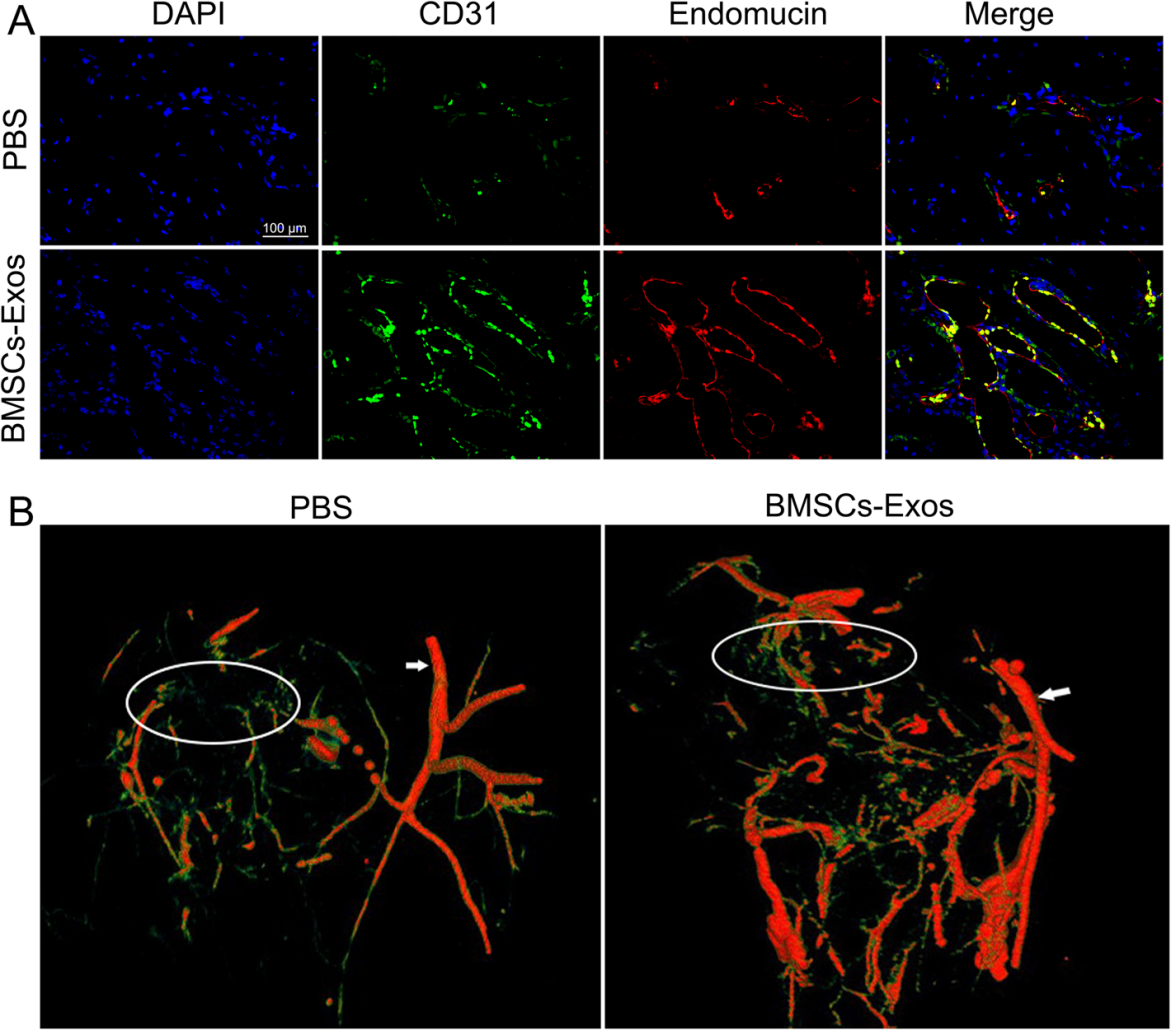
Effects of BMSC-Exos on the vessels around the rotator cuff insertion in rats. **a** BMSC-Exos promoted the expression of CD31 and endomucin around the tendon-bone interface 4 weeks after reconstruction, as determined by immunofluorescence (*n* = 9/group). Scar bar 100 μm. **b** BMSC-Exos promoted angiogenesis around the rotator cuff 4 weeks after reconstruction, as determined by angiography (*n* = 9/group) (white circle, rotator cuff insertion; white arrow, axillary artery)

The blood supply around the tendon-bone interface of the rotator cuff and tendon revascularization are extremely important for tendon-bone healing [26, 27]. To verify the effect of BMSC-Exos on angiogenesis in vivo, BMSC-Exos or PBS was injected into the tail vein of rats after rotator cuff reconstruction to observe the effect of BMSC-Exos on angiogenesis around the tendon-bone interface. Recently, CD31 and endomucin have been reported to play an important role in angiogenesis and promote tendon-bone healing [28, 29]. We observed the expression of CD31 and endomucin around the tendon-bone interface with immunofluorescence and found that BMSC-Exos promoted the expression of CD31 and endomucin (Fig. 4a). Next, we verified the effect of BMSC-Exos on angiogenesis in rats. After rotator cuff reconstruction, BMSC-Exos was injected into the tail vein in rats. Angiogenesis around the rotator cuff was observed by angiography 4 weeks after reconstruction. BMSC-Exos injection was found to have a stronger effect than PBS (Fig. 4b). The angiogenic results confirmed that BMSC-Exos promoted angiogenesis around the rotator cuff endpoint after rotator cuff reconstruction in rats.

### BMSC-Exos inhibited the secretion of proinflammatory factors after rotator cuff reconstruction in vivo

**Fig. 5:**
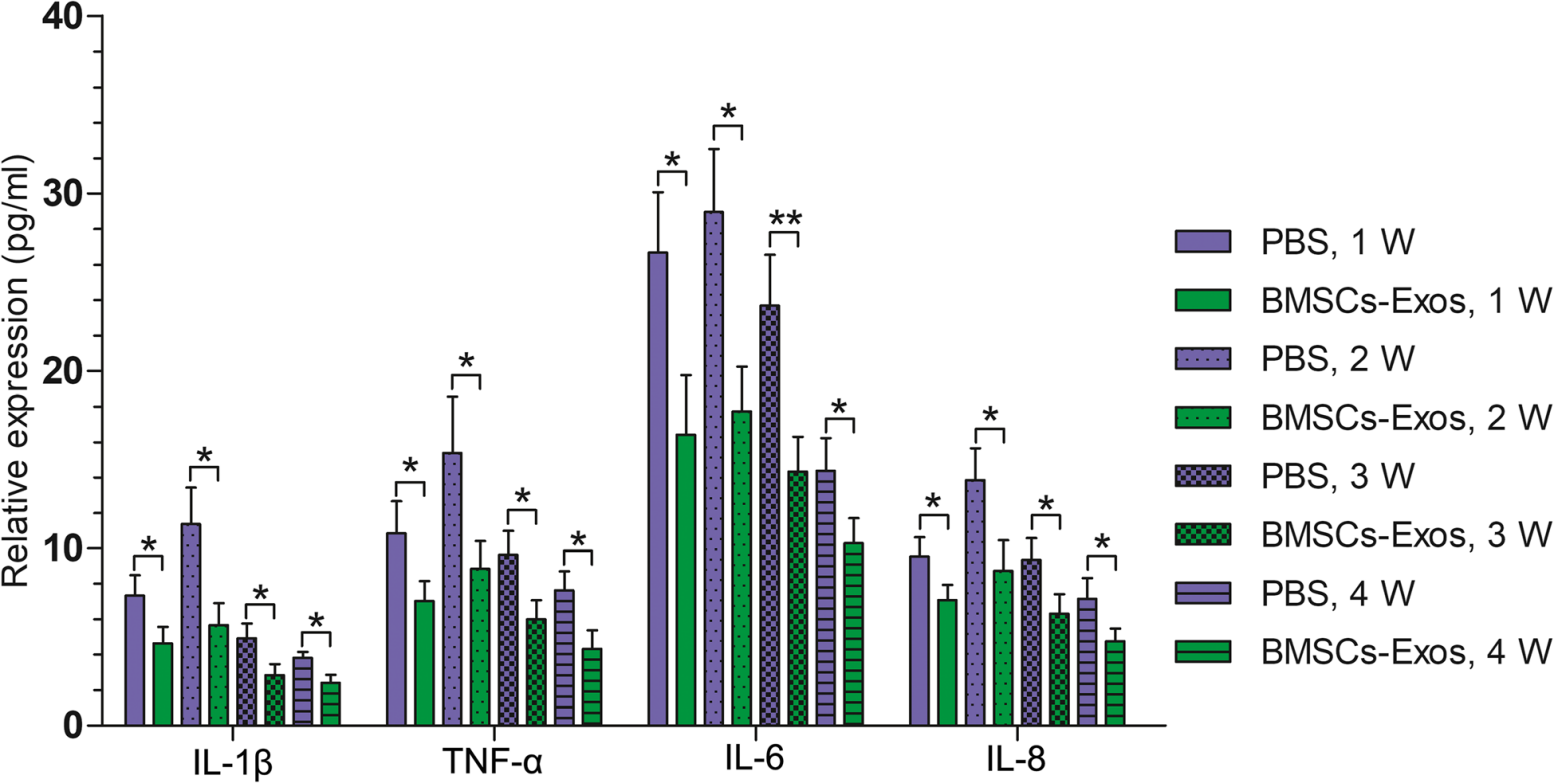
BMSC-Exos reduced the expression of IL-1β, TNF-α, IL-6, and IL-8 in rat serum (each experiment was independently repeated three times)

Since an inflammatory response is adverse to tendon-bone healing, the effect of proinflammatory factors such as tumor necrosis factor-α (TNF-α), IL-1β, IL-6, and IL-8 should be suppressed as much as possible in the early postoperative period [30–34]. We injected BMSC-Exos at 0, 1, 2, and 3 weeks after reconstruction, and ELISA was used to detect the levels of proinflammatory factors in the serum of rats at 1, 2, 3, and 4 weeks after reconstruction. It was found that BMSC-Exos significantly reduced the serum levels of TNF-α, IL-1β, IL-6, and IL-8 in rats at all time points (Fig. 5).

### BMSC-Exos inhibited M1 polarization of macrophages

**Fig. 6:**
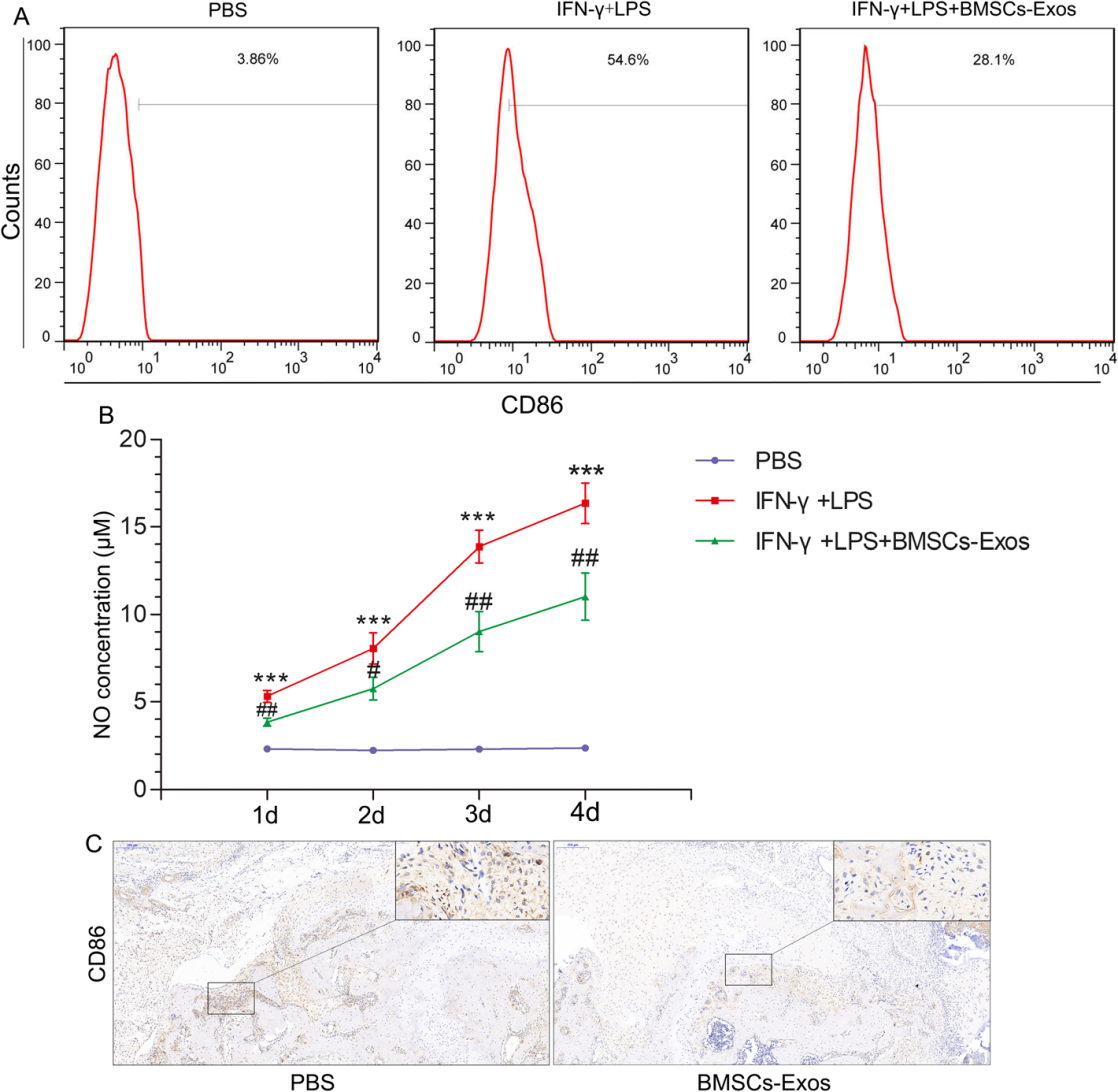
Effects of BMSC-Exos on the polarization of M1 macrophages. **a** BMSC-Exos inhibited the expression of the surface marker CD86 in M1 macrophages (each experiment was independently repeated three times). **b** BMSC-Exos inhibited the release of NO by M1 macrophages (each experiment was independently repeated three times). **c** BMSC-Exos inhibited the expression and distribution of M1 macrophages around the tendon-bone interface 2 weeks after reconstruction (*n* = 9/group). *Compared with the PBS group. ^#^Between the LPS and BMSC-Exos groups, ^#^*p* < 0.05; ^##^*p* < 0.01

Since BMSC-Exos inhibited the secretion of proinflammatory factors related to tendon-bone healing in rats, we hypothesized that BMSC-Exos could affect M1 macrophage secretion of proinflammatory factors. BMSC-Exos was used in M1 macrophage polarization of U937 cells. The flow cytometry results show that the expression of CD86, a surface marker of M1 macrophages, was decreased (Fig. 6a), confirming that the polarization of M1 macrophages was inhibited. These results suggest that BMSC-Exos may inhibit the release of proinflammatory factors by inhibiting the polarization of M1 macrophages.

Activation of M1 macrophages increases the expression of inducible nitric oxide synthase, thus promoting the production of a large amount of NO [35]. We tested the effect of BMSC-Exos on NO release during polarization induction of M1 macrophages. The results show that the addition of BMSC-Exos (100 μg/mL) to the culture solution inhibited NO release during IFN-γ and LPS induction of M1 macrophages. The NO release by the IFN-γ and LPS + BMSC-Exos groups was higher than the IFN-γ and LPS + PBS groups, confirming that BMSC-Exos inhibited M1 macrophage polarization of U937 cells (Fig. 6b).

We observed the expression and distribution of M1-type macrophages around the tendon-bone interface in response to PBS or BMSC-Exos 2 weeks after rotator cuff reconstruction. Immunohistochemistry showed that the expression and distribution of M1-type macrophages around the tendon-bone interface were lower in the BMSC-Exos group than in the PBS group. This result indicates that BMSC-Exos inhibited the M1-type polarization of macrophages around the tendon-bone interface (Fig. 6c).

### BMSC-Exos inhibited the release of related inflammatory factors by M1 macrophages

**Fig. 7:**
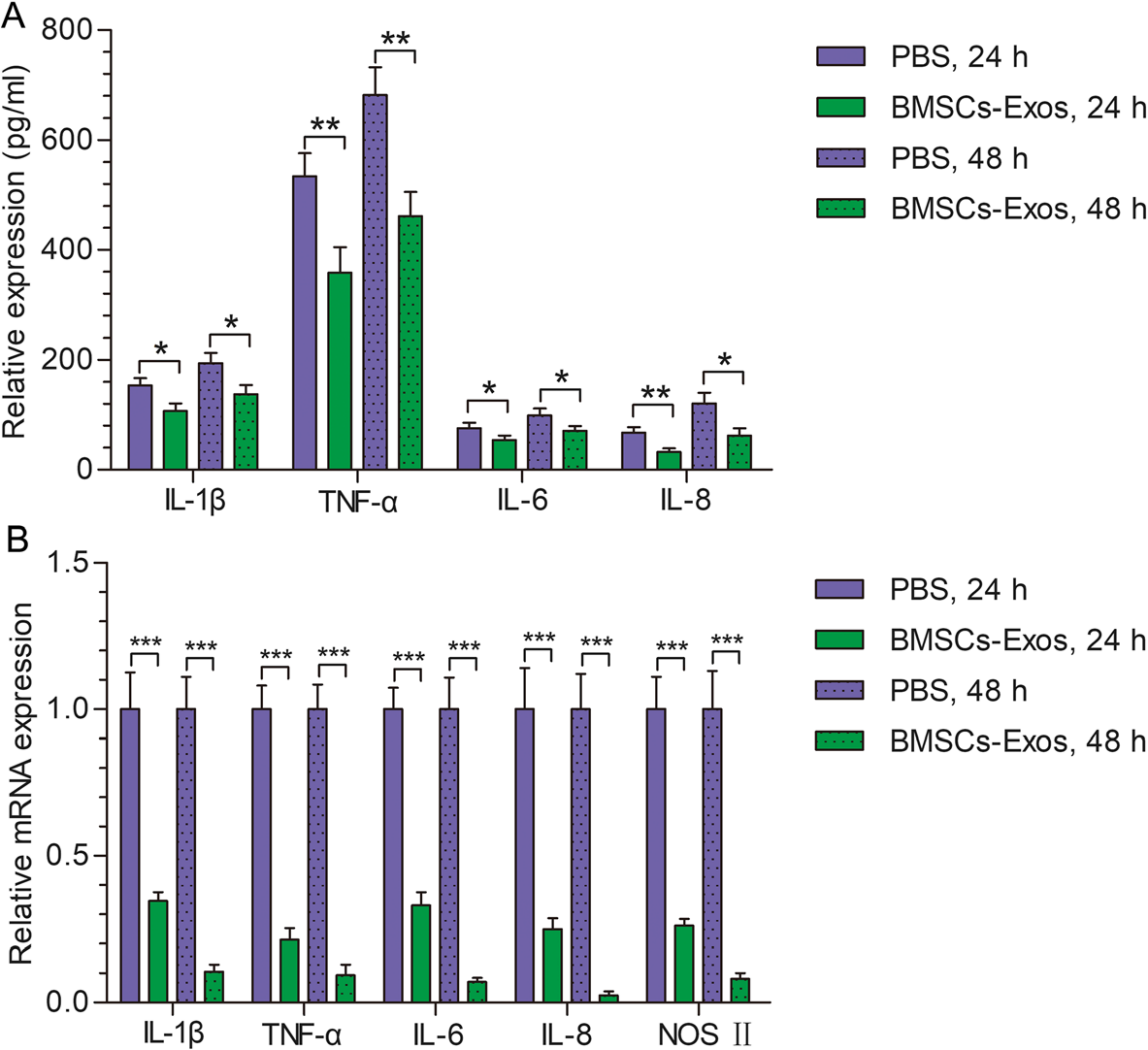
Effects of BMSC-Exos on M1 macrophage-related inflammatory factors. **a** BMSC-Exos inhibited the secretion of inflammatory factors by M1 macrophages. **b** BMSC-Exos inhibited mRNA expression of M1 macrophage-associated inflammatory factors. Each experiment was independently repeated three times

We further studied the effect of BMSC-Exos on M1 macrophages. After adding BMSC-Exos (100 μg/mL) or PBS for 24 h and 48 h, the ELISA results show that TNF-α, IL-1β, IL-6, and IL-8 in M1 macrophages were lower in the BMSC-Exos group than in the PBS group. These results confirm that BMSC-Exos inhibited the release of proinflammatory factors by M1 macrophages (Fig. 7a). The mRNA expression of related proinflammatory factors was detected by qRT-PCR. Compared with the PBS group, TNF-α, IL-1β, IL-6, IL-8, and NOS-2 gene expression was decreased in the BMSC-Exos group (Fig. 7b).

### Mechanical and biological effects of BMSC-Exos on rotator cuff reconstruction in rats

**Fig. 8:**
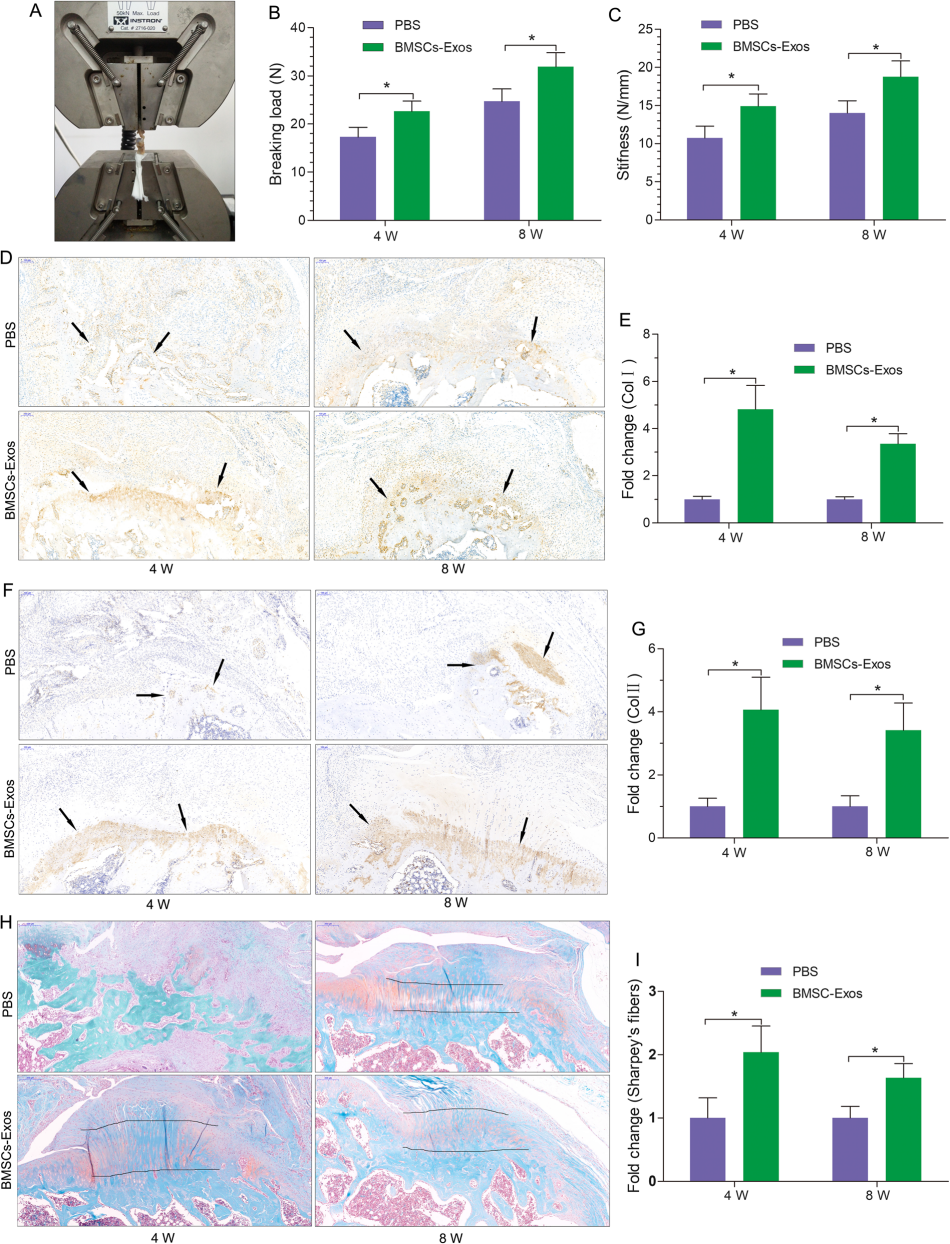
Mechanical and biological effects of BMSC-Exos on rotator cuff reconstruction in rats. **a** Biomechanical test of the reconstruction rotator cuff (supraspinatus). **b**, **c** BMSC-Exos promoted the maximum breaking load and stiffness at 4 and 8 weeks (*n* = 9/group). **d** BMSC-Exos promoted the Col I expression of the tendon-bone interface at 4 and 8 weeks (black arrows; *n* = 9/group). Scar bar 100 μm. **e** Quantitative analysis of **d**. **f** BMSC-Exos promoted Col II expression at the tendon-bone interface at 4 and 8 weeks (black arrows; *n* = 9/group). Scar bar 100 μm. **g** Quantitative analysis of **f**. **h** BMSC-Exos promoted the expression of Sharpey’s fibers and proteoglycan at the tendon-bone interface at 4 and 8 weeks (between two black lines; *n* = 9/group). Scar bar 200 μm. **i** Quantitative analysis of Sharpey’s fibers in **h**

Tendon-bone complex rupture occurs mostly at the tendon-bone interface. In this experiment, the maximum breaking load and stiffness were selected as the biomechanical test parameters. The maximum breaking load was the maximum force that the tendon could bear, and the stiffness was the linear slope of the load-displacement curve, which could reflect the difficulty of elastic deformation of the tissue. Four weeks after rotator cuff reconstruction in rats, the molecular and biological effects in the tendon-bone interface had recovered, and the healing was relatively solid at 8 weeks [36]. Therefore, we selected 4 weeks and 8 weeks as the two time points. Our results show that there were significant differences in the maximum breaking load and stiffness between the two groups at 4 weeks and 8 weeks after the reconstruction and that the BMSC-Exos group was significantly superior to the PBS group (Fig. 8a–c).

We observed the healing of the tendon-bone interface at 4 and 8 weeks after reconstruction and found that the growth of the tendon-bone interface in the BMSC-Exos group was better than in the PBS group. The tendon-bone interface contains four layers of tissue: fibrous connective tissue, noncalcified fibrous cartilage, calcified fibrous cartilage, and bone tissue. The fibrous connective tissue layer includes mainly Col I, while the noncalcified and calcified fibrous cartilage layers include mainly Col II [37]. We observed Col I and Col II expression at the tendon-bone interface at 4 and 8 weeks after reconstruction. We found that Col I and Col II expression in the BMSC-Exos group were significantly higher than in the PBS group (Fig. 8d–g). Sharpey’s fibers dominated the scar tissue between the tendon and the bone after the rotator cuff reconstruction [4]. We found that Sharpey’s fibers in the BMSC-Exos group were denser than those in the PBS group at 4 and 8 weeks after reconstruction (Fig. 8h, i). The fibrocartilage layer present on the tendon-bone interface is associated with the degree of tendon-bone healing. Safranin-Fast Green staining was used to detect the proteoglycan expression level at the tendon-bone interface at 4 and 8 weeks. Proteoglycan expression was lower, and the density of the tendon-bone interface was smaller in the PBS group than in the BMSC-Exos group (Fig. 8h).

## Discussion

The area where the tendon and ligament attach to the bone is named the tendon-bone interface and is an important connection between the soft tissue and the bone. The tendon-bone interface can be easily damaged, at which time it needs repair. The tendon-bone healing process requires the proliferation and differentiation of different types of cells in close coordination. The quality of tendon-bone healing directly affects the probability of a re-tear after the rotator cuff repair. At present, the normal tissue structure of the tendon-bone interface cannot be repeated after a rotator cuff reconstruction. After rotator cuff reconstruction, the scar tissue between the tendon and bone is composed mainly of Sharpey’s fibers. This causes the tensile strength of the rotator cuff insertion point after reconstruction to be significantly reduced compared with the normal insertion point [38].

At present, tendon-bone healing is a difficult and hot topic in sports medicine. Stem cells and tissue engineering have made some progress in tendon-bone healing. BMSCs have a variety of proliferation and differentiation potential and are widely used to treat bone and cartilage defects. They promote meniscus regeneration and healing, and they also repair muscle tendon lesions [39]. In addition, BMSCs can regulate local growth factors and cytokines to promote wound repair and tissue regeneration [40, 41]. BMSC-derived exosomes regulate the tissue microenvironment, promote tissue repair and reconstruction, and perform a wide range of biological functions. Based on the role of BMSCs in regulating the immunity and metabolism of various cells through exosomes [42], it is reasonable to speculate that BMSC-Exos could be involved in the regulation of tendon-bone healing.

Many studies have suggested that the blood supply is extremely important for tendon-bone healing and that tendon revascularization can promote tendon-bone healing [43, 44]. Demirag et al. [45] showed that a higher degree of vascularization resulted in more mature histomorphology of the tendon-bone interface, which contained more Sharpey’s fibers. Yoshikawa et al. [27] found that in the early stage of anterior cruciate ligament reconstruction, failure of graft revascularization led to surgical failure if the angiogenesis-triggering mechanism was missing. Takayama et al. [46] found that inhibiting VEGF expression at the tendon-bone interface inhibited angiogenesis and reduced tendon maturity and biomechanical strength. They also found that overexpression of VEGF hindered improvement in grafted tendon strength. Some studies have suggested that vascular growth is detrimental to tendon-bone healing, but, in fact, most of these studies concluded that vascular growth is associated with degenerative tendinopathy [47]. Fealy et al. [48] found that blood supply increased early after rotator cuff reconstruction and then decreased gradually over time. The best site of blood supply was around the tendon, while the worst site of blood supply was at the fixation point or bone groove. Fealy et al. believed that increased blood supply at the fixation point or bone groove could improve the quality of tendon-bone healing. Our view is similar to the meaning of Fealy et al. We consider tendon-bone healing from a “plain” perspective and that a lack of blood supply leads to slow tissue repair. We think that promoting blood supply around the tendon-bone interface, rather than vascular ingrowth to the internal tendon-bone interface, would promote tendon-bone healing.

Firstly, we observed in vitro that BMSC-Exos promoted the proliferation, migration, and angiogenic tube formation of HUVECs and that BMSC-Exos activated the VEGF and Hippo signaling pathways. Recent studies have shown that VEGFR phosphorylation led to the decreased phosphorylation of LATS and YAP and that YAP entered the nucleus and activated the Hippo signaling pathway, strongly promoting angiogenesis [49]. We found that activation of the VEGF and Hippo signaling pathways by BMSC-Exos may be independent of each other. Activation of the Hippo signaling pathway by BMSC-Exos was not entirely dependent on the VEGF signaling pathway, indicating that BMSC-Exos have extensive and active effects in promoting angiogenesis. Subsequently, we demonstrated in vivo that BMSC-Exos promoted angiogenesis around the tendon-bone interface.

In the early stages of tendon-bone healing (inflammatory exudation phase), inflammatory factors, such as TNF-α, IL-1β, IL-6, and IL-8, promote the progress of acute inflammation, restrain the directional differentiation of MSCs, block the secretion of extracellular matrix proteoglycans, and slow the tendon-bone healing [30–34]. Therefore, we speculated that BMSC-Exos could inhibit the production of proinflammatory factors and promote tendon-bone healing. The role of inflammatory factors in tendon-bone healing is complex. Their expression in the tendon-bone healing process peaks at about 1~2 weeks after surgery, remains elevated for a few weeks, and then returns to baseline levels 16 weeks after surgery [50]. Considering that the molecular biology effect of the rat rotator cuff tendon-bone interface reaches basic recovery 4 weeks after reconstruction [36], we conducted rat caudal vein injections of BMSC-Exos at four time points: 0, 1, 2, and 3 weeks after reconstruction. The results show that after injections of BMSC-Exos, the levels of TNF-α, IL-1β, IL-6, and IL-8 were decreased at 1, 2, 3, and 4 weeks after reconstruction. These results confirm that BMSC-Exos could inhibit the production of proinflammatory factors in rats.

A few days after reconstruction of the rotator cuff, macrophages are recruited that release various inflammatory mediators at the reconstructed place. This induces an inflammatory response that leads to the formation of scar tissue [51]. Macrophages are classified into M1 and M2 macrophages. After induction, M1 macrophages can secrete a large number of proinflammatory factors, participate in antigen presentation, and accelerate the development of inflammation. Dagher et al. [52] showed that postoperative immobilization can effectively reduce macrophage aggregation, the inflammatory response, and scar tissue formation and, thus, promote tendon-bone healing. Hays et al. [53] demonstrated that liposomal clodronate can inhibit the expression of macrophages and thus promote tendon-bone healing. However, there is still a lack of relevant clinical studies, and further experimental studies are needed.

We speculated that BMSC-Exos could inhibit the production of proinflammatory factors by affecting M1 macrophage polarization. BMSC-Exos antagonized the in vitro polarization of U937 cells into M1 macrophages by IFN-γ and LPS. In vivo experiments also found that BMSC-Exos inhibited the distribution of M1 macrophages around the tendon-bone interface. We further speculated that BMSC-Exos could have the same effect on M1 macrophages. When BMSC-Exos were added to the culture medium of M1 macrophages, the proinflammatory factors secreted by the M1 macrophages were reduced. These results confirm that BMSC-Exos can inhibit the secretion of proinflammatory factors by M1 macrophages. Therefore, BMSC-Exos not only inhibit the polarization of M1 macrophages but also interfere with the function of M1 macrophages related to the secretion of proinflammatory factors.

**Fig. 9:**
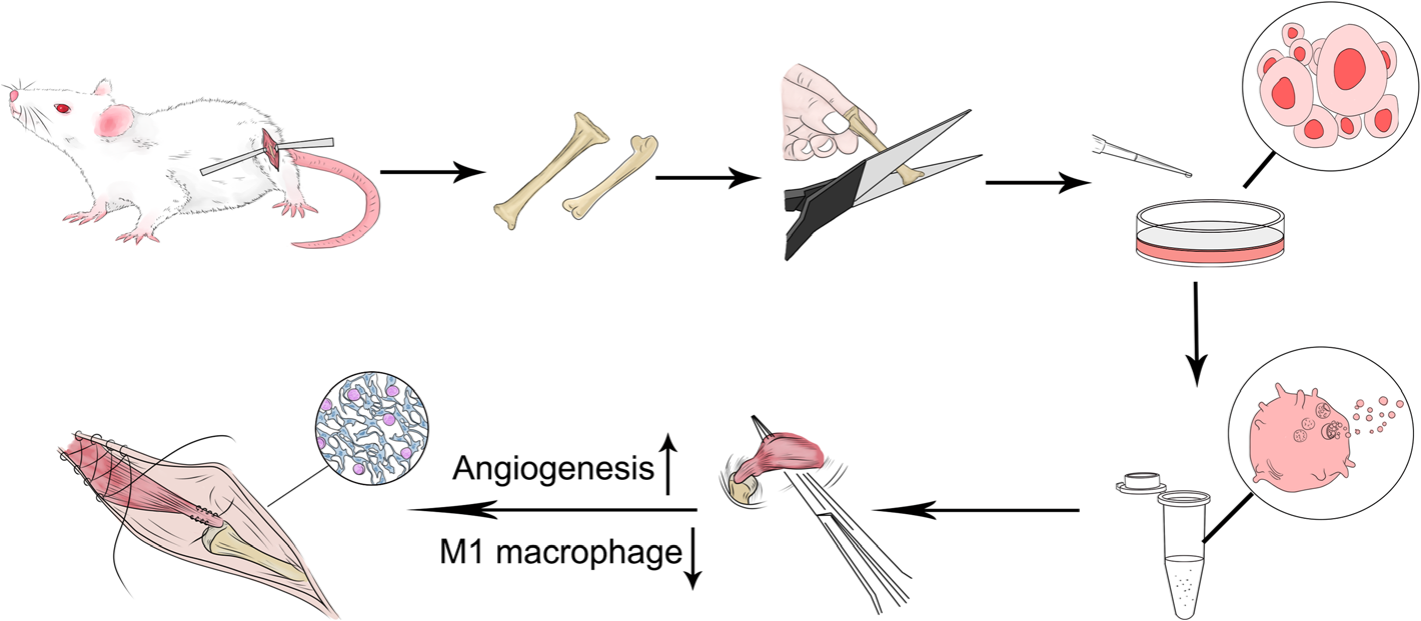
BMSC-Exos promote tendon-bone healing after rotator cuff reconstruction by promoting angiogenesis and inhibiting M1 macrophage in rats

Finally, BMSC-Exos were injected into the tail vein of rats after rotator cuff reconstruction to observe the mechanical and biological effects of exosomes on rotator cuff reconstruction. The results confirm that BMSC-Exos increased the breaking load and stiffness after rotator cuff reconstruction. The histological observation on the tendon-bone interface confirms that BMSC-Exos promoted tendon-bone healing. Tendon-bone healing is a complex repair and healing process involving multiple factors. Single-factor or target studies cannot perfectly explain or solve the complex process of tendon-bone healing. Exosomes contain plenty of substances, such as RNA, DNA, proteins, enzymes, and lipids, that possess various functions. We believe that exosomes with multiple functions may be an effective solution to the complex process of tendon-bone healing. Due to the diversity and complexity of the components in BMSC-Exos, we are currently unable to determine the most critical substances promoting tendon-bone healing, which is a limitation of this study. In general, BMSC-Exos greatly promote tendon-bone healing (Fig. 9).

## Conclusion

In summary, BMSC-Exos promote tendon-bone healing after rotator cuff reconstruction in rats. BMSC-Exos achieve this by promoting angiogenesis through the VEGF and Hippo signaling pathways. They also suppress inflammation by inhibiting M1 macrophage polarization and M1 macrophage secretion of proinflammatory factors.

## Supplementary Information

**Additional file 1:** **Fig. S1.** Identification of BMSCs. **a** Cell morphology of BMSC observed by optical microscope. b BMSCs exhibited a characteristic spindle-like morphology. **c, d,** and **e** BMSCs showed potential differentiation capacity for osteogenesis, adipogenesis and chondrogenesis.

**Additional file 2:** **Fig. S2.** Flow cytometric analysis of characteristic BMSC cell surface markers (CD34, CD73, CD90, and CD105).

**Additional file 3:** **Fig. S3.** Flow cytometric analysis of characteristic BMSC-Exos surface markers (CD63, CD81, and CD9).

## Data Availability

The data that support the findings of this study are available from the corresponding author upon reasonable request.

## Abbreviations

RCTs: Rotator cuff tears
MSCs: Mesenchymal stem cells
BMSC-Exos: Bone MSC-derived exosomes
HUVECs: Human umbilical vein endothelial cells
RCT: Rotator cuff tear
SD: Sprague-Dawley
Col I: Type I collagen
Col II: Type II collagen
qRT-PCR: Quantitative real-time polymerase chain reaction
ELISA: Enzyme-linked immunosorbent assay
CCK-8: Cell Counting Kit-8
EdU: 5-Ethynyl-29-deoxyuridine
NO: Nitric oxide
TEM: Transmission electron microscope
WB: Western blot
VEGF: Vascular endothelial growth factor
VEGFR: Vascular endothelial growth factor receptor
LATS: Large tumor suppressor serine/threonine protein kinases
YAP1: Yes-associated protein
DMSO: Dimethyl sulfoxide
TNF-α: Tumor necrosis factor-α
IL: Interleukin
IFN-γ: Interferon-gamma
LPS: Lipopolysaccharide
PBS: Phosphate-buffered saline
