# Supplementary figures and images for: Bone marrow mesenchymal stem cell-derived exosomes promote rotator cuff tendon-bone healing by promoting angiogenesis and regulating M1 macrophages in rats
Source: Stem Cell Res Ther. 2020 Nov 25;11:496. doi: 10.1186/s13287-020-02005-x (PMC7687785; doi:10.1186/s13287-020-02005-x)

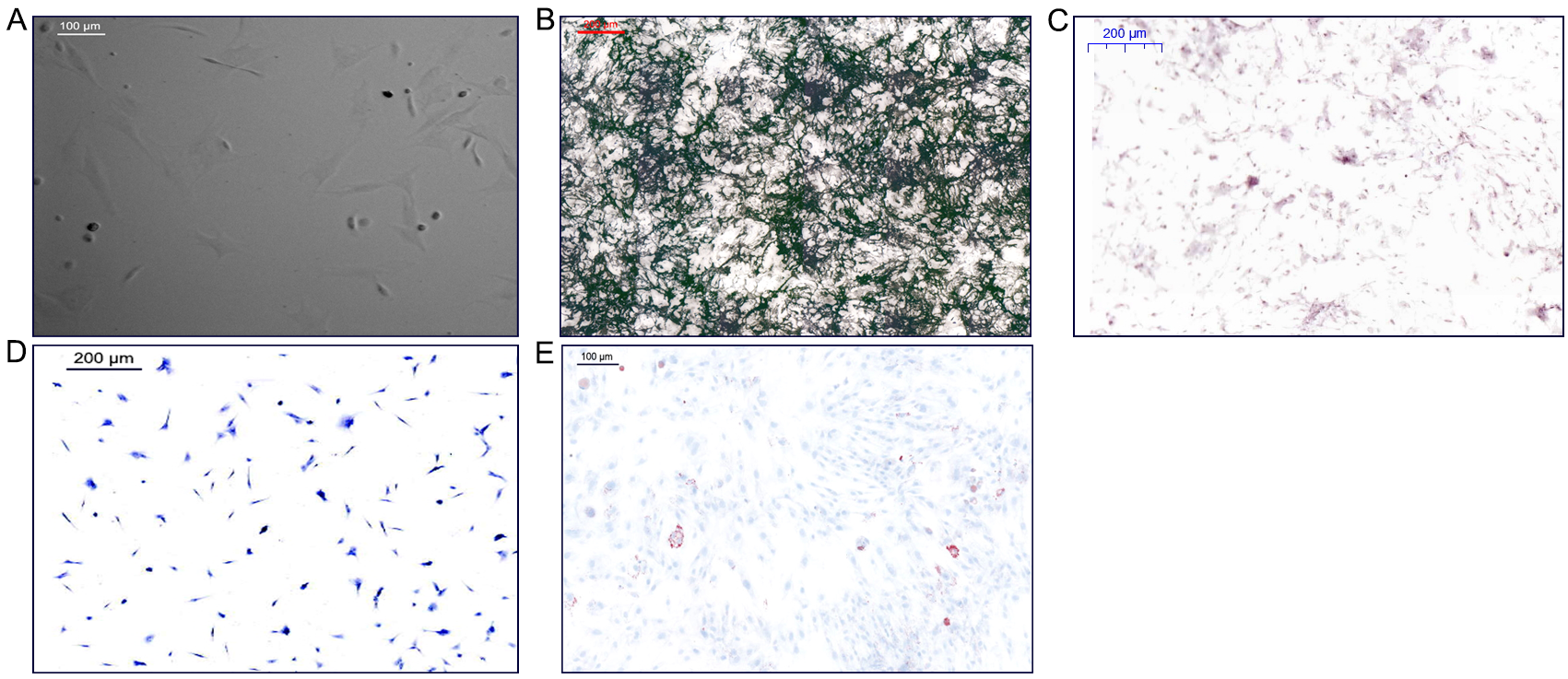

Supplement: Supplementary file 1 — Additional file 1: Fig. S1. Identification of BMSCs. a Cell morphology of BMSC observed by optical microscope. b BMSCs exhibited a characteristic spindle-like morphology. c, d, and e BMSCs showed potential differentiation capacity for osteogenesis, adipogenesis and chondrogenesis. [file 13287_2020_2005_MOESM1_ESM.tif]

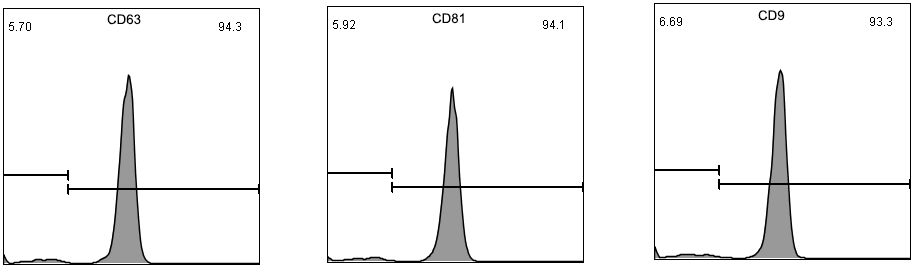

Supplement: Supplementary file 2 — Additional file 2: Fig. S2. Flow cytometric analysis of characteristic BMSC cell surface markers (CD34, CD73, CD90, and CD105). [file 13287_2020_2005_MOESM2_ESM.tif]

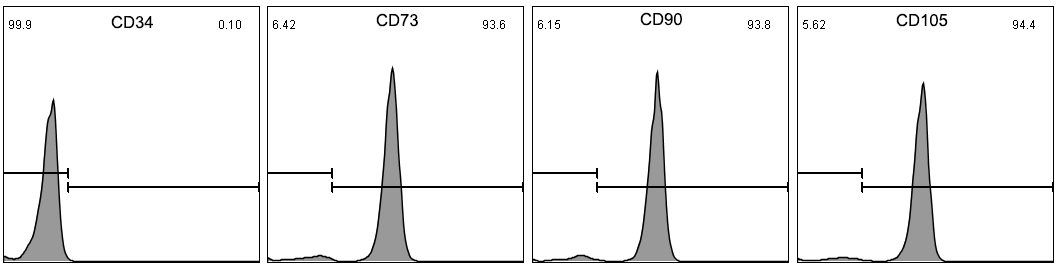

Supplement: Supplementary file 3 — Additional file 3: Fig. S3. Flow cytometric analysis of characteristic BMSC-Exos surface markers (CD63, CD81, and CD9). [file 13287_2020_2005_MOESM3_ESM.tif]
